# Risk of Infection and Sepsis in Pediatric Patients with Traumatic Brain Injury Admitted to Hospital Following Major Trauma

**DOI:** 10.1038/s41598-018-28189-0

**Published:** 2018-06-28

**Authors:** Anjli Pandya, Kathleen Helen Chaput, Andrea Schertzer, Diane Moser, Jonathan Guilfoyle, Sherry MacGillivray, Jaime Blackwood, Ari R. Joffe, Graham C. Thompson

**Affiliations:** 1 Department of Emergency Medicine, Cumming School of Medicine, University of Calgart, Calgary, AB Canada; 2 Alberta Children’s Hospital Research Institute, Cumming School of Medicine, University of Calgary, Calgary, AB Canada; 3 Alberta Trauma Services, Alberta Health Services, Edmonton, AB Canada; 4 Data Integration, Measurement and Reporting, Alberta Health Services, Calgary, AB Canada; 5 Department of Pediatrics, Cumming School of Medicine, University of Calgary, Calgary, AB Canada; 6 Department of Pediatrics, University of Alberta, Edmonton, AB Canada

## Abstract

Head injury accounts for 29% of all traumatic deaths in children. Sepsis is significantly associated with an increased risk of mortality in adult traumatic brain injury patients. In the pediatric population, this relationship is not well understood. The objective of this study was to compare the proportion of pediatric traumatic brain injury (TBI) patients and trauma patients without brain injury (NTBI) who developed sepsis or any infection during their index hospital admission. We performed a retrospective study of all trauma patients <18 years of age, admitted to trauma centres in Alberta, Canada from January 1, 2003 to December 31, 2012. Patients who died within 24 hrs of trauma (n = 147) and those with burns as the primary mechanism of injury (n = 53) were excluded. Hospital admission data for the remaining 2556 patients was analyzed. 1727 TBI patients and 829 NTBI patients were included. TBI was associated with lower odds of developing sepsis (OR 0.32 95% CI 0.14–0.77 p = 0.011). TBI was not found to be independently associated with developing any infectious complication after adjusting for confounding by Injury Severity Score (OR 1.25 95% CI 0.90–1.74 p = 0.180). These relationships warrant further study.

## Introduction

Head injury is an important cause of morbidity and mortality in pediatric trauma patients. The prevalence of head injury in the general population under twenty-five years of age has been estimated at 30% based on North American and New Zealand cohort studies, with an incidence rate of 1.10–2.36 per 100 per year^1^. Head injury accounts for 29% of all traumatic deaths in the pediatric population based on United States of America data^2^.

The association between infection and trauma has been well established in the adult literature. Sepsis occurs in 2% of adult trauma patients overall, and up to 75% in those with severe traumatic brain injury. Adult trauma patients who develop sepsis are at significantly higher risk of death compared with those who do not^3,4^. Adult studies have also shown an association between sepsis and mortality in traumatic brain injury patients, and demonstrated that the elevated risk of death from sepsis persists beyond one-year post-injury in traumatic brain injury survivors compared with matched controls^5^.

In the pediatric population, the relationship between trauma, brain injury and sepsis is not as well understood. Bell *et al*. reported an overall infection rate of 19% in a cohort of 212 pediatric trauma patients, and determined that cerebral edema or subarachnoid hemorrhage was a risk factor for infection (OR 6.8 95% CI 2.2–21.3)^6^; however, patients with traumatic brain injury were not explicitly compared to trauma patients without traumatic brain injury, and the primary objective of the study was to determine the incidence of infection in the general pediatric trauma population.

An improved understanding of the relationship between traumatic brain injury, infection and sepsis in pediatric patients is necessary to ensure that pediatric trauma victims are optimally managed both in hospital and after discharge. Healthcare providers can benefit from improved knowledge of potential complications in the course of traumatic brain injury recovery, and ultimately patient outcomes can be optimized. This study aimed to explore the patterns of sepsis and infection in pediatric traumatic brain injury patients through a direct comparison of the proportion of pediatric traumatic brain injury (TBI) patients and trauma patients without brain injury (NTBI) who developed sepsis during the index hospital admission. The secondary objectives of the study were to compare the proportion of TBI and NTBI patients who developed any infectious complication, as well as to describe the demographics, clinical characteristics and mortality of pediatric TBI patients.

## Results

### Population

A total of 2556 patients meeting the inclusion criteria were identified over a 10-year period from January 1, 2003 to December 31, 2012. The overall mean Injury Severity Score (ISS) of the cohort was 22 (SD 8.7). 1727 (67.6%) patients suffered TBI; 719 (41.6%) were isolated TBI (ITBI), and 1008 (58.4%) were non-isolated TBI (ATBI). 829 (32.4%) patients did not have any traumatic brain injury. TBI patients had significantly higher mortality (4.5% vs 0.4% p < 0.001) than NTBI patients (Tables 1, 2). There were significantly more intubated patients in the TBI cohort versus the NTBI cohort (35.7% vs 13.8% p < 0.001).

**Table 1:** Baseline comparison of traumatic brain injury and non-traumatic brain injury patients.

Baseline Characteristic	TBI				NTBI (n = 829)	Overall Cohort (n = 2556)
	All TBI (n = 1727)	ATBI (n = 1008)	ITBI (n = 719)	Unadjusted p-value		
Age, mean (SD)	10 (6.1)	12 (5.1)	8 (6.4)	<0.001	13 (4.4)	11 (5.7)
Males, n (%)	1103 (63.9)	638 (63.3)	465 (64.7)	0.56	581 (70.1)	1684 (65.9)
Blunt trauma, n (%)	1704 (98.7)	998 (99.0)	207(28.8)	<0.001	753 (90.8)	2457 (96.1)
Penetrating trauma, n (%)	23 (1.33)	10 (0.99)	13 (1.81)	0.87	76 (9.17)	99 (3.87)
Length of Stay (days), mean (SD)	10 (20)	12 (20.9)	8 (18.3)	<0.001	9 (12.5)	10 (17.9)
Intubated, n (%)	617 (35.7)	434 (43.1)	183 (25.5)	<0.001	114 (13.8)	731 (28.6)
ICU Stay, n (%)	741 (42.9)	489 (48.5)	252 (35.1)	<0.001	223 (26.9)	964 (37.7)
Length of Mechanical Ventilation at Trauma Centre in Intubated Patients (days), mean (SD)	5.4 (8.7)	5.8 (6.9)	5.1 (12.1)	0.57	5.7 (13.3)	5.5 (9.6)
ISS, mean (SD)	23 (9.3)	25 (10.8)	19 (4.4)	<0.001	20 (6.7)	22 (8.7)
GCS at Scene, mean (SD)	11 (4.4)	11 (4.3)	11 (4.5)	0.92	14 (2.2)	12 (4.2)
GCS at Trauma Centre, mean (SD)	14 (2.6)	13 (2.8)	14 (2.3)	<0.001	15 (0.81)	14 (2.2)
Operative Intervention, n (%)	663 (38.4)	454 (45.0)	209 (29.1)	<0.001	356 (42.9)	1019 (39.9)

TBI- Traumatic Brain Injury, ATBI – Associated with TBI; ITBI – Isolated Traumatic Brain Injury; NTBI- No Traumatic Brain Injury, ICU- Intensive Care Unit, ISS- Injury Severity Score, GCS-Glasgow Coma Scale

All rounded to nearest whole number.

**Table 2:** Outcomes of pediatric trauma patients.

Outcome	TBI (n = 1727)		NTBI (n = 829)	Overall Cohort (n = 2556)	Unadjusted p-value (TBI vs NTBI)
	ATBI (n = 1008)	ITBI (n = 719)			
Infection, n (%)	187 (10.8)		55 (6.6)	242 (9.5)	p = 0.001
	131 (13.0)	56 (7.8)			
Sepsis. n (%)	9 (0.5)		13 (1.6)	22 (0.9)	p = 0.007
	8 (0.8)	1 (0.1)			
Death, n (%)	77 (4.5)		3 (0.4)	80 (3.1)	p < 0.001
	48 (4.8)	29 (4.0)			

TBI- Traumatic Brain Injury, ATBI- Associated with Traumatic Brain Injury, ITBI- Isolated Traumatic Brain Injury, NTBI- No Traumatic Brain Injury.

### Sepsis and Infection Outcomes

A total of 22 (0.9%) cases of sepsis (9 TBI – 8 ATBI, 1 ITBI; 13 NTBI) were identified (Supplementary Table 1). Overall 242 (10.8%) patients developed at least one infectious complication (187 TBI-131 ATBI, 56 ITBI; 55 NTBI) during the index admission. The overall incidence of sepsis and of infection in this cohort was 0.86% and 9.5% respectively. The primary sites of infectious complications are outlined in Table 3.

**Table 3:** Primary site of infectious complications.

Primary Anatomic Site	TBI with Any Infection (n = 187)	NTBI with Any Infection (n = 55)	Unadjusted p-value
Respiratory	104 (55.6)	17 (30.9)	0.001
CNS	6 (3.2)	0	0.18
Intra-Abdominal	12 (6.4)	2 (3.6)	0.44
Genitourinary	49 (26.2)	11 (20)	0.35
Soft Tissue/Bone	29 (15.5)	10 (18.2)	0.64
Other/Unspecified	78 (41.7)	34 (61.8)	0.009

TBI- Traumatic Brain Injury, NTBI- No Traumatic Brain Injury, CNS- Central Nervous System.

TBI was found to be significantly associated with lower odds of sepsis (OR 0.32 95% CI 0.14–0.77 p = 0.011) (Table 4). There was no significant association between TBI and odds of infection after adjusting for confounding by ISS (OR 1.25 95% CI 0.90–1.74 p = 0.001) (Table 4). Using reverse-elimination modeling, age, sex, operative intervention, invasive non-operative intervention, and duration of mechanical ventilation did not have a significant confounding effect.

**Table 4:** Results of Logistic Regression Analysis.

Variable	Odds Ratio (95% Confidence Interval)	p-value	Pseudo R-squared
**Odds of Any Infectious Complication**			
Traumatic Brain Injury	1.25 (0.90–1.74)	0.180	0.103
Injury Severity Score	1.08 (1.06–1.09)	>0.001	
**Odds of Sepsis**			
Traumatic Brain Injury	0.32 (0.14–0.77)	0.011	0.026

### Association of infection and intubation

Intubated patients were examined post-hoc. There were 617 intubated TBI patients and 114 intubated NTBI patients. There was no significant difference in overall infection in intubated TBI patients versus intubated NTBI patients (24.6% vs 27.2% p = 0.56). There was no significant difference in respiratory infections between intubated TBI patients and intubated NTBI patients (10.5% vs 14.8% p = 0.23).

### Association of infection and mortality

The overall mortality in this cohort was 3.1% (80/2556). There was no significant difference in mortality between patients who developed an infection and those who did not (Table 2), nor in those who developed sepsis and those who did not. Table 2 outlines mortality in children with (ATBI + ITBI) and without traumatic brain injury.

## Discussion

We have evaluated the incidence of sepsis and infection in children with TBI enrolled in a province-wide trauma registry. In our cohort of children admitted to hospital for trauma-related illness (minimum ISS = 12), we found the overall incidence of sepsis and any infectious complication was low (0.86%, 9.5% respectively). The infection rate in our cohort are consistent with those previously reported in children (9–19%)^5^ and lower than that of adults which historically ranges from 16–80%^5^.

Contrary to previous reported findings in the adult trauma population that an independent relationship exists between TBI and sepsis, we identified reduced odds of sepsis between TBI and NTBI patients in our pediatric cohort. Selassie *et al*. determined sepsis was an independent risk factor for in-hospital death in adult patients with TBI (HR 1.34 p < 0.001)^7^. Harrison-Felix *et al*. described the causes of death of adult TBI patients surviving at least 1 year post-injury in a retrospective cohort study, and found sepsis to be a significantly more common cause of death compared to matched controls without TBI (Standardized mortality ratio 11.6 for death from sepsis.)^5^. Molecular studies also support a relationship between TBI and immune function; *ex-vivo* immune studies in trauma patients who develop late onset septic shock have demonstrated altered expression of human leukocyte antigen-DR (HLA-DR) and reduced CXC- chemokine receptor 1 induced activity^8^. Neonatal studies have demonstrated altered levels of IL-6, IL1-beta and TNF-alpha in neonates with white matter damage and sepsis^9^. These studies indicate a complex neuro-immune interaction, which may be the basis of any relationship between sepsis, infection and TBI. As such, the apparent association of TBI with lower odds of sepsis in this study warrants further study, specifically to delineate why pediatric patients may experience an association in the opposite direction to their adult counterparts, and to determine whether TBI is truly associated with a lower odds of sepsis in pediatric patients.

Despite the decreased odds of sepsis in TBI patients, no significant independent association was found between overall infection and TBI, after adjusting for confounding by ISS (OR 1.25 95% CI 0.9–1.74, p = 0.18). This may be because infections included were not limited to infection at the primary site of trauma, or because TBI impacts immune function, including its response to infectious episodes.

The mortality of TBI patients surviving at least 24 hours (4.5%) found in our cohort is consistent with prior studies that estimate the rate of in-hospital death in pediatric TBI patients to be 2.5–5%^10^. No significant difference in mortality was present between patients with infection or sepsis as compared to patients without infectious complications, however the small number of infectious outcomes, and low mortality, limits the power of this study to detect a significant mortality difference. We did not perform an in-depth mortality analysis, as this was not the primary objective of our study and any identified predictors of mortality based on this cohort (selected to study the relationship between TBI and infection) may not be representative of overall risk factors for mortality in pediatric trauma patients.

## Limitations

Our study is limited by the administrative source of our data, and the retrospective design. Though the ATR collects data on a large number of variables, this data is coded by multiple different trained analysts, across a large number of sites; the reliability of coding cannot be determined. The study definitions of TBI, ITBI, NTBI, sepsis and infection relied on diagnostic coding. Due to the province-wide nature of the study, individual health records could not be accessed to confirm these classifications, especially sepsis or infection, and TBI based on neuroimaging, or other gold-standard measurement. This may have resulted in a misclassification of some sepsis cases as non-sepsis cases; moreover, patients meeting clinical criteria for sepsis may not have been coded as sepsis but rather by primary site of infection.

Additionally, ICD coding for infection relays little information about the clinical importance of an identified infection. DIMR data is coded based on chart documentation from discharge.

summaries, progress notes and admission notes; however, coders are unable to rely on or interpret clinical data including lab results, meaning an ICD code for ‘sepsis’ requires a definitive written mention of sepsis in the chart. This is a well-described challenge in sepsis literature^11^. Similarly, without patient-level chart data, we were unable to determine the number of infectious complications per patient or the time to onset of infection from admission.

Misclassification resulting from these diagnostic-coding errors is, however, estimated to be non-differential (i.e. occurring in the TBI patients as commonly as the NTBI patients), and thus would bias our results toward the null. Additionally, we estimate the magnitude of any misclassification bias to be small, as trained medical professionals that operate according to province-wide standards enter the ATR data. Therefore we estimate that if our results are biased, they would be slightly underestimating the magnitude of the true relationship between sepsis, infection and TBI. Additionally, although our sample size is relatively large, our outcome of pediatric sepsis is rare, and thus we were limited in the numbers of variables we were able to explore in our regression models, and this may have limited the goodness of fit of our final models. Future studies with larger samples are needed to further explore these associations. Finally, our study was limited to short-term clinical outcomes of children and did not address the potential long-term relationship between TBI and infection demonstrated in previous adult studies.

## Conclusions

We have identified a complex relationship between TBI, infection and sepsis in pediatric patients and highlighted the need for further study. TBI is associated with lower odds of sepsis, but has no significant association with the development of general infection. Further studies are required to confirm this finding, and examine the biologic mechanisms underlying this effect.

## Methods

### Study Setting and Population

**Figure 1:**
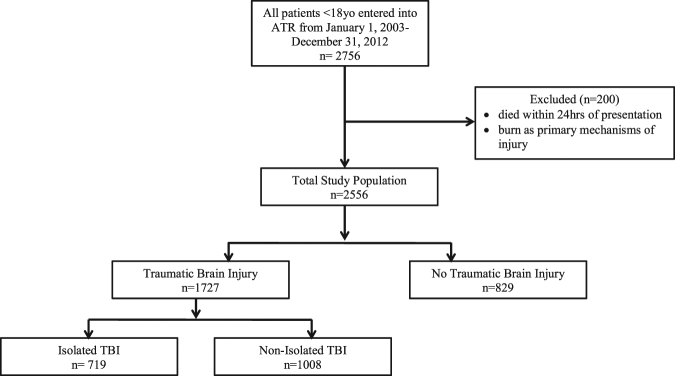
Application of inclusion and exclusion criteria to generate study population. *ATR – Alberta Trauma Registry*, *TBI - Traumatic Brain Injury*.

We performed a retrospective study of all pediatric trauma patients, under 18 years of age, admitted to any level I-III trauma centres in Alberta, Canada over a 10-year period, using an administrative trauma database. The Alberta Trauma Registry (ATR) is a province-wide electronic registry, managed by Alberta Health Services and administered and overseen by the Provincial Trauma Committee, including all patients with an injury severity score (ISS) > 12.

who have been treated (either primarily or after transfer) at a Level I-III trauma centre (TC) in Alberta. The level of trauma care provided at a given TC in Alberta is designated in accordance with the Trauma Association of Canada Accreditation Guidelines^12^. The ISS is a 0–75 point scoring scale that is calculated based on the three highest abbreviated injury scale (AIS) scores; a higher ISS score indicates a more severe injury^13^. The catchment area of TCs in Alberta includes areas of western Saskatchewan, eastern British Columbia as well as the North West Territories. All injury-related admissions, and emergency department resuscitations at each TC are reviewed and entered into the ATR by trained data analysts. We extracted data from the records of all patients <18 years of age who were entered into the ATR from January 1, 2003 to December 31, 2012. Patients who died within 24hrs of presentation to the emergency department (ED) were excluded (n = 147), as they would have been unlikely to develop a new infection within that period of time. Patients with ‘burns’ listed as the primary mechanism of injury were also excluded (n = 53) due to the established relationship between burns and infection, and the low probability of concurrent TBI. The remaining 2556 patients were included in our sample and analysis (see Fig. 1).

### Data acquisition and Management

From the ATR database we collected demographics, ISS, abbreviated injury scale (AIS) scores, procedural intervention, disposition and mortality data. Hospital admission data related to the index trauma visit were obtained from the Alberta Health Services (AHS) Data Integration, Measurement and Reporting (DIMR) service, and linked to the ATR data using provincial health-care number, date of birth and date of injury. The DIMR database provided the diagnostic codes assigned to each admission. The Conjoint Health Research Ethics Board of the University of Calgary approved this study (REB13-0189); all methods were performed in accordance with the relevant guidelines and regulations.

### Measures

Our exposure variable for this study was traumatic brain injury (TBI). We used the Centers for Disease Control (CDC) Data Systems Case Definition of TBI^8^, excluding ICD-9-CM code 804, to identify cases of TBI. ICD-9-CM code 804 refers to fractures of the skull OR face^9^. As we were unable to obtain clinical records to confirm the injuries, we excluded code 804 in order to eliminate the possibility of including isolated facial trauma patients. Patients with TBI were identified from the ATR dataset by having a non-zero AIS score to the head/neck/c-spine region and an ICD-9-CM code^14^ corresponding to TBI (800, 801, 803, 850–854) (see supplemental digital content- appendix 1). The AIS is a scale that grades injury to each body region on a scale from 0 to 6, where 1 is a ‘mild’ injury and 6 is an ‘unsurvivable’ injury^15^. Conversely, patients with no traumatic brain injury (NTBI group) were identified by having an AIS score of 0 to the head/neck/c-spine region OR an AIS score >=1 to the head/neck/c- spine region but no ICD-9- CM code corresponding to TBI. TBI patients were further categorized into isolated TBI (ITBI) and non-isolated TBI (ATBI) patients based on AIS scores. ITBI patients were identified as.

Having an ICD-9-CM code of 800, 801, 803 and/or 850–854 AND no AIS score present in the chest/T-spine, abdomen/L-spine, extremities/pelvis, or external regions. If a patient had only TBI injuries plus facial injuries (but no injuries to any other body region including the c-spine region) they were included as ITBI patients.

The outcomes of interest were infectious complications, and sepsis, which were identified using ICD-10 codes^10^ from the linked DIMR data (see supplemental digital content- appendix 1).

Infectious complications were not limited to infections at the primary site of trauma, since the theorized link between brain injury and infection involves altered immune-modulatory function and this effect should apply universally to all forms of infection^15,16^. Infections were classified by primary anatomic site based on ICD-10 codes (see supplemental digital content- appendix 1). ‘Respiratory’ infections were defined involving the upper aerodigestive tract, lower airways, pulmonary parenchyma or pleural space. ‘Central Nervous System’ (CNS) infections were defined as involving the brain, spinal cord, CSF or meninges. ‘Intra-abdominal’ infections were defined as involving any intra-abdominal organ, excluding those of the genitourinary tract, which were classified separately as ‘Genitourinary’ infections. ‘Skin/Soft-Tissue’ infections were classified as involving any layer of the skin, myofascial planes, or bone. Infections involving sites other than those previously listed, or cases in which an anatomic site was not coded, were classified as ‘Other/Unspecified’. 24 patients had dual records in the ATR (48 records) because they were admitted to more than one hospital for the same injury. The data contributing to each admission were used in the analysis, and were controlled for dependence.

### Data Analysis

All data were analyzed using STATA v13.1. Descriptive statistics were reported for demographic variables, trauma mechanism-related variables, and various clinical outcomes (ICU stay, death etc). Univariable analyses were then conducted to assess for associations between potential confounding variables and the exposure and outcome variables. Categorical variables were compared using Pearson’s chi-square analysis, and continuous variables were compared using Student’s t-test, at an alpha level of 5% for parametric data. Mann-Whitney rank-sum testing was used for non-parametric data at the same alpha level. Multivariable logistic regression was performed to model the odds of sepsis, and the odds of infection separately, with exposure to TBI. Variables initially explored in the analysis to control for potential confounding were: ISS score, age (years), sex, Glasgow Coma Scale (GCS), invasive non-operative interventions (Y/N), intensive care unit admission (Y/N), length of mechanical ventilation (days), penetrating mechanism of injury (Y/N), and operative intervention (Y/N). We followed a rule of thumb in which the number of variables in the logistic regression is at most 10% of the minimum of absent or present outcomes which limited our models to a maximum of 2 explanatory variables. A reverse-elimination process of modeling was employed, wherein the maximum number of potential confounders is included in initial models, and insignificant and non-confounding variables are selectively removed until only significant and confounding variables remain^16^.

### Data Availability

The datasets generated during and analyzed during the current study are available from the corresponding author on reasonable request.

## Electronic supplementary material

Supplementary Information
