# Supplementary material for: Risk of Infection and Sepsis in Pediatric Patients with Traumatic Brain Injury Admitted to Hospital Following Major Trauma
Source: Sci Rep. 2018 Jun 28;8:9798. doi: 10.1038/s41598-018-28189-0 (PMC6023879; doi:10.1038/s41598-018-28189-0)
Supplement: Supplementary file 1 — Supplementary Information [file 41598_2018_28189_MOESM1_ESM.pdf]

Supplementary Information – Definitions and Coding

Title of Manuscript: Risk of Infection and Sepsis in Pediatric Patients with Traumatic Brain Injury Admitted to Hospital Following Major Trauma

Authors: Anjli Pandya MD FRCPC, Kathleen Helen Chaput PhD, Andrea Schertzer BSc. MPH, Diane Moser CHIM, Jonathan Guilfoyle MD FRCPC , Sherry MacGillivray RN BN, Jaime Blackwood MD FRCPC, Ari R Joffe MD FRCPC and Graham C. Thompson MD FRCPC

Supplementary Information – Definitions and Coding

Traumatic Brain Injury Definitions & ICD Code Application

The following ICD-9-CM[i] codes were used to identify traumatic brain injury patients from the Alberta Trauma Registry, as detailed under "Variables and Definitions" section:

800	Fracture of vault of skull
801	Fracture of base of skull
803	Other and unqualified multiple fractures of the skull
850	Concussion
851	Cerebral laceration and contusion
852	Subarachnoid, subdural and extradural hemorrhage, following injury
853	Other and unspecified intracranial hemorrhage, following injury
854	Intracranial injury of other and unspecified nature

Infection Definitions & ICD Code Application

Cases of infection and sepsis were identified based on diagnostic coding entered into the Alberta Health Services Data Integration, Measurement and Reporting (DIMR) dataset, which allows up to twenty-five diagnostic codes per admission, and were grouped by primary infection site. By convention, 'primary' anatomic infection site was defined as the site included in the first diagnostic code that had an anatomic specification. As an example, if patient X had four diagnostic codes entered and both the 3rd and 4th code included an anatomic specification, the site specified by the 3rd code would be designated the 'primary' infection site.

For brevity, the broad ICD-10[i] classifications used for each definition are included, but a full list of detailed specifiers is excluded, in this appendix.

The following conditions were applied within each category of ICD-10 codes to individual specifiers:

- Inflammatory conditions that were not specified as being infectious in etiology (eg. 'Nonpyogenic meningitis') were not included as infections.
- Only acute conditions were included (i.e. chronic conditions such as 'chronic meningitis' were excluded).

Respiratory Infections

Respiratory infections were defined as those infections that involved the nasopharynx, upper airway tract, the lower airways, lung parenchyma, pleural space, or are pathophysiologically thought to arise from one of the above areas.

J00-J06	Acute upper respiratory tract infections
J09-J18	Influenza and pneumonia
J20-J22	Other acute lower respiratory infections
J85	Abscess of lung and mediastinum
J86	Pyothorax
H66	Suppurative otitis media
H70	Mastoiditis and related conditions
All codes within 'I. Certain Infectious and Parasitic Diseases' (A00-B99) that met the above defined anatomic requirements	

Supplementary Information – Definitions and Coding

Central Nervous System (CNS) Infections
CNS infections were defined as those infections that involved the brain parenchyma, spinal cord, cerebrospinal fluid, meninges or meningeal spaces.

G00-G03	Meningitis (Bacterial, Classified elsewhere, In other infectious and parasitic disease)
G04-G05	Encephalitis, myelitis and encephalomyelitis
G06-G07	Intracranial and intraspinal abscess
All codes within 'I. Certain Infectious and Parasitic Diseases' (A00-B99) that met the above defined anatomic requirements	

Intra-Abdominal Infections
Intra-abdominal infections included all infections with a primary anatomic site located within the abdomen but excluding the genitourinary tract and perianal area

K35	Acute appendicitis
K67	Disorders of peritoneum in infectious diseases classified elsewhere
A00-A09	Intestinal infectious disease
K83	Cholangitis
K81	Cholecystitis
K75	Abscess of liver
All codes within 'I. Certain Infectious and Parasitic Diseases' (A00-B99) that met the above defined anatomic requirements	

Genitourinary Infections
Genitourinary infections included those that involved the kidneys, ureterus, bladder, urethra or reproductive organs (internal and external).

N30	Cystitis
N70-N77	Inflammatory diseases of female pelvic organs
N29	Other disorder of kidney and ureter in infectious and parasitic disease classified elsewhere
N45	Orchitis and epididymitis
N48-N49	Other disorders of penis
All codes within 'I. Certain Infectious and Parasitic Diseases' (A00-B99) that met the above defined anatomic requirements	

Soft Tissue and Bone Infections
Soft tissue and bone infections included those that involved the epidermis, dermis, subcutaneous tissue, muscle, fascial planes, connective tissue or bone and surrounding layers.

L00-L08	Infections of the skin and subcutaneous tissue
T84.5	Infection due to internal joint prosthesis
M86	Osteomyelitis
M72.6	Necrotizing fasciitis
M71.0-M71.1	Abscess or other infective bursitis
K61	Abscess of anal regions
All codes within 'I. Certain Infectious and Parasitic Diseases' (A00-B99) that met the above defined anatomic requirements	

Other or Unspecified Infections
All other infectious complications, excluding sepsis (see below), which did not meet any of the definitions of all other listed categories were grouped as 'Other or Unspecified' infections.

T814	Infection following a procedure not elsewhere specified
H44	Disorders of globe (Purulent endopthalmitis or other infective endophlthamitis)
I31	Other diseases of pericardium
I33, I38, I39	Acute endocarditis (Valve specified, Valve not specified, Classified elsewhere)
All codes within 'I. Certain Infectious and Parasitic Diseases' (A00-B99) that did not meet specified anatomic requirements, or had no anatomic specification	

Sepsis Definition & ICD Code Application

A40	Streptococcal sepsis
A41	Other sepsis
R57.2	Septic shock
R65.0-R65.1	Systemic Inflammatory Response Syndrome of infectious origin (with and without organ failure)

Invasive Non-Operative Interventions
Non-operative interventions entered into the ATR were divided into 'Invasive' and 'Non-Invasive' non-operative interventions, based on the primary author's impression of the infection risk associated with each intervention.

Invasive Non-Operative Interventions	Non-Invasive Non-Operative Interventions
Tracheostomy	Bag mask ventilation
Cricothyroidotomy	Oropharyngeal or nasopharyngeal airway insertion
Intra-osseus infusion	Laryngeal mask airway insertion
Central line insertion	Combitube insertion
Chest tube placement	Peripheral intravenous placement
Needle decompression	Chest compressions
Venous cutdown	Cardioversion
Emergency department thoracotomy	Defibrillation
Intracranial pressure monitor	C-spine immobilization
External ventricular drain	Computed tomography
Burr hole	Splinting/Casting
Diagnostic peritoneal lavage	Ultrasound
Pericardiocentesis	Magnetic Resonance Imaging
Blood product administration	Closed reduction
Halo traction/Traction pins	Echocardiogram
Major suturing	

[i] Classifications of Diseases, Functioning and Disability [Internet]: National Centre for Health Statistics [updated June 2013, reviewed June 2013, cited September 14, 2015]. Available from: http://www.cdc.gov/nchs/icd.htm

TABLE S1: Individual characteristics of patients who developed sepsis

Patient	Age	Sex	TBI Status	Mechanism of Injury	ISS	Intubated	LOS	Post ED Location	Operative Intervention	Infection Site	ICU	Death
**26***	13	M	NTBI	Penetrating	25	Yes	117	OR	Yes	Soft Tissue &Other	Yes	No
**66**	15	M	NTBI	Blunt	18	No	52	ICU	Yes	Other	Yes	No
**211**	14	F	ATBI	Blunt	45	Yes	20	ICU	No	Respiratory & other	Yes	No
**307**	3	M	ITBI	Blunt	20	Yes	9	ICU	No	Other	Yes	No
**381**	17	M	ATBI	Blunt	50	Yes	51	OR	Yes	Respiratory & Soft Tissue	Yes	No
**533**	16	M	NTBI	Blunt	16	No	11	Ward	Yes	Other	No	No
**586**	14	M	NTBI	Blunt	25	Yes	32	OR	Yes	Other	Yes	No
**663**	2	M	NTBI	Blunt	34	Yes	50	OR	Yes	Soft Tissue & other	Yes	No
**744**	5	M	NTBI	Burn	25	Yes	53	ICU	Yes	Other	Yes	No
**1003**	11	M	NTBI	Penetrating	17	Yes	30	OR	Yes	Soft Tissue & Other	Yes	No
**1060**	17	M	ATBI	Blunt	35	Yes	7	ICU	Yes	Respiratory	Yes	Yes
**1070**	4	M	NTBI	Penetrating	25	Yes	19	OR	Yes	Other	Yes	No
**1652**	15	F	ATBI	Blunt	59	Yes	66	ICU	Yes	Respiratory	Yes	No
**1880**	4	M	NTBI	Blunt	26	Yes	61	ICU	Yes	Other	Yes	No
**1946**	12	M	NTBI	Blunt	16	No	18	ICU	Yes	Other	Yes	No
**2048**	17	F	ATBI	Blunt	29	Yes	16	ICU	Yes	Respiratory & Genitourinary	Yes	No
**2082**	7	M	NTBI	Blunt	16	Yes	32	OR	Yes	Other	Yes	No
**2181**	16	F	ATBI	Blunt	27	Yes	15	OR	Yes	Respiratory	Yes	No
**2295**	17	F	NTBI	Blunt	26	Yes	53	OR	Yes	Respiratory	Yes	No
**2333***	14	F	ATBI	Blunt	57	Yes	35	OR	Yes	Soft Tissue	Yes	No
**2454**	3	M	NTBI	Blunt	17	Yes	36	Ward	Yes	Other	Yes	No

**Patients were entered into the trauma registry more than once because they were admitted to more than one hospital for the same injury, and thus counted as independent encounters as different procedures and treatments may have been delivered at different hospitals. In this table, data are amalgamated for each of these patients.*
